# Mood disorder in a patient with a benign thalamic cystic lesion: a case report

**DOI:** 10.1186/1752-1947-7-107

**Published:** 2013-04-17

**Authors:** Pallavi Dham, Jacob Alexander

**Affiliations:** 1 Rural and Remote Mental Health Service, Glenside, South Australia, Australia

**Keywords:** Anti-convulsant mood stabilizers, Anti-psychotic medication, Cystic lesions, Cognition, Mood disorder, Thalamus

## Abstract

**Introduction:**

The thalamus is increasingly gaining importance in psychiatric disorders. There are case reports in the literature of neuropsychiatric symptoms associated with thalamic infarcts. The present report elucidates the complexities of linking neuropsychiatric symptoms to a benign thalamic brain lesion, and its impact on management.

**Case presentation:**

We present the case of a Caucasian man in his early 30s, who presented with a difficult to treat bipolar illness and coexisting thalamic lesion.

**Conclusions:**

In this report we explore the possible links between our patient’s symptoms and his brain lesion. We discuss the possible neuronal mechanisms that may be involved and debate the most appropriate management strategies. We hope this report will assist further insights into the role of the thalamus in psychiatric disorders.

## Introduction

The scientific literature documents a number of neurobehavioral symptoms associated with vascular and benign thalamic lesions [1-3]. We report the case of a man with symptoms suggestive of bipolar illness and a coexisting left midbrain-thalamic benign cystic lesion [4], highlighting the complexities of attributing psychiatric symptoms to an abnormal brain structure and the associated challenges in choosing appropriate management.

## Case presentation

A 30-year-old Caucasian man was admitted to our adult psychiatry in-patient unit in September 2011 with uncontrolled impulsive behaviors while on medications for previously diagnosed bipolar illness. The symptom cluster leading to admission included preoccupation with a venue for adult entertainment, social disinhibition (relieving himself in public) and irresponsible behavior (leaving his shop unattended), leading to a significant impact on his functioning, relationships and associated loss of prestige. There were no features of goal-directed hyperactivity, racing thoughts or a decreased need for sleep. Coexisting was a benign brain lesion incidentally identified before the onset of the mood disorder on a routine imaging study performed following a motor vehicle accident. The only obvious physical deficit was a progressively worsening distal tremor of his right upper limb.

He was diagnosed with a major depressive episode nine years ago in the context of a relationship breakup and associated substance use (alcohol, cannabis). He was treated with escitalopram for two years. Seven years later his diagnosis was changed to bipolar disorder following an episode of mania with psychotic symptoms. Adequate symptom control was not achieved while he was treatment compliant on a combination of valproate (up to 2g/day) and atypical anti-psychotics (olanzapine, then ziprasidone up to 80mg/day) in the absence of substance use.

## Discussion

We grappled with the issue of a possible relationship between the space-occupying lesion and our patient’s psychiatric symptoms. Additionally we debated the management of our patient’s condition.

**Figure 1:**
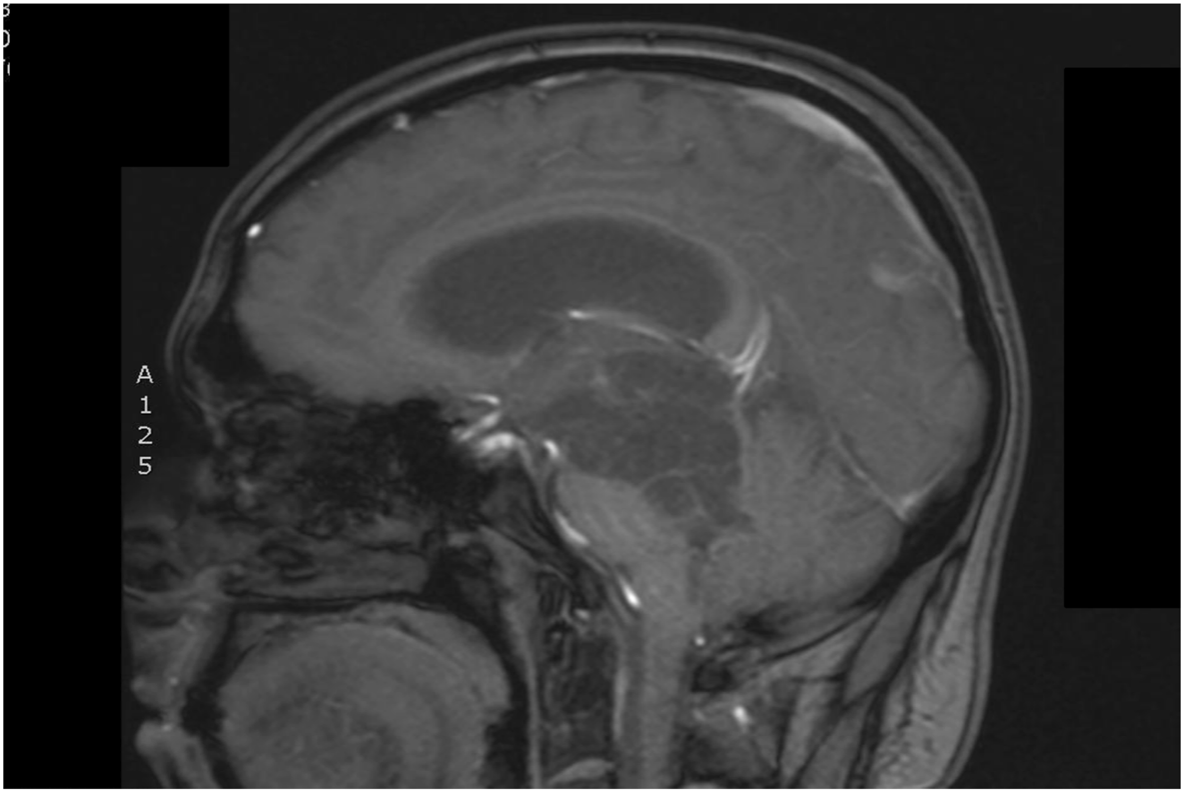
Magnetic resonance image of the brain showing the complex cystic lesion extending from the left hemipons to the thalamus.

On admission all medications were tapered and ceased over two weeks to enable a drug-free period of observation. Unlike the persistent symptoms seen in mood disorders, episodic impulsive behaviors were noted in clear sensorium, lasting for minutes to hours with subsequent sketchy recall. Our patient was appropriate and insightful in between. No psychotic features were noted. Cognitive deficits in recall and orientation on the Mini-Mental State Examination (MMSE; 28 out of 30), and concrete responses and impaired verbal fluency on the Frontal Assessment Battery (12 out of 18) were noted. Neuropsychological testing showed deficits in auditory-visual recall, executive function, verbal fluency and abstract verbal reasoning, hallmarks of thalamic impairment, particularly with regard to the dorsolateral pre-frontal circuit with minimal impairment of general intellectual functioning. The results of a neurological examination revealed mild weakness of the left third cranial nerve, right-sided upper motor neuron deficits, wing-beating tremor and cerebellar signs, suggestive of midbrain pathology. The results of a complete blood count, serum electrolyte tests, renal and liver function tests, serum glucose test and urine microscopy study were all unremarkable. A urine drug screen was negative. A magnetic resonance imaging (MRI) scan performed on admission showed a complex cystic lesion in the left half of the brain stem extending from the left lower hemipons to the left thalamus, increased in size in comparison to the initial scan (Figure 1).

An electroencephalogram (EEG) revealed bilateral theta activity, often seen in deep midline lesions. However, our patient was also medicated with chlorpromazine and drowsy; both conditions can display this symptom pattern and hence this was non-conclusive.

Following the evaluation he was started on monotherapy with carbamazepine, gradually titrated to a dose of 500mg/day. He continues to maintain improvement at six-month follow-up. Neurosurgical advice discouraged surgical intervention owing to high risk of an adverse outcome related to the location of the lesion and a poor risk-benefit ratio.

The thalamo-frontal circuits are implicated in mood and psychotic disorders [5]. Thalamo-cortical dysrhythmia (TCD) has attracted increasing attention as an underlying mechanism for psychiatric disorders [6]. In TCD, persistent thalamic delta and/or theta range activity serves as the trigger for thalamo-cortical dysfunction [7]. We hypothesize that TCD possibly underpinned our patient’s behavioral disturbances and subtle personality changes. Though there were symptoms suggestive of mania, it was an atypical presentation as the mood was not persistently elevated and our patient’s behaviors were episodic. Another atypical feature was the lack of an adequate explanation of his experiences by our patient himself. We believe that the psychiatric symptoms were largely related to the brain lesion and not merely coexistent.

TCD has been proposed to be related to the dysfunction of low-voltage calcium channels. Anti-convulsants such as valproate and ethosuximide are known to act on low-voltage calcium channels. Carbamazepine acts primarily via blocking of voltage-gated sodium channels and may act on other channels including calcium channels [8]. The concurrent use of anti-psychotic medications may have interfered with the initial treatment with sodium valproate in this instance, plausibly explaining the poor response.

## Conclusions

Psychiatric symptoms related to benign brain lesions can be particularly difficult to diagnose because of their gradual onset and insidious course. This particular case highlights the role of the thalamus in behavioral symptoms. Patients with atypical and even typical psychiatric symptoms with co-existing brain lesions need closer examination to delineate the extent of their linkage, with far-reaching implications on management. Atypical anti-psychotics, though popular in the management of bipolar disorder, may be detrimental in such cases.

## Consent

Written informed consent was obtained from the patient for publication of this case report and any accompanying images. A copy of the written consent is available for review by the Editor-in-Chief of this journal.

## Competing interests

All authors declare that they have no competing interests.

## Authors’ contributions

According to the definition given by the International Committee of Medical Journal Editors (ICMJE), both authors qualify for authorship based on making substantial contributions to the intellectual content and drafting of the manuscript. Both authors read and approved the final manuscript.
